# Graded Empathy: A Neuro-Phenomenological Hypothesis

**DOI:** 10.3389/fpsyt.2020.554848

**Published:** 2020-11-24

**Authors:** Jonathan Levy, Oren Bader

**Affiliations:** ^1^Department of Neuroscience and Biomedical Engineering, Aalto University, Espoo, Finland; ^2^Baruch Ivcher School of Psychology, Interdisciplinary Center Herzliya, Herzliya, Israel; ^3^Department of General Psychiatry, Center of Psychosocial Medicine, University of Heidelberg, Heidelberg, Germany

**Keywords:** empathy, neurophenomenology, magnetoencephalography (MEG), intergroup conflicts, cognitive empathy, affective empathy, empathy dichotomy, phenomenology

## Abstract

The neuroscience of empathy has enormously expanded in the past two decades, thereby making instrumental progress for the understanding of neural substrates involved in affective and cognitive aspects of empathy. Yet, these conclusions have relied on ultrasimplified tasks resulting in the affective/cognitive dichotomy that was often modeled and overemphasized in pathological, developmental, and genetic studies of empathy. As such, the affective/cognitive model of empathy could not straightforwardly accommodate and explain the recent surge of neuroscientific data obtained from studies employing naturalistic approaches and intergroup conditions. Inspired by phenomenological philosophy, this article paves the way for a new scientific perspective on empathy that breaks thorough the affective/cognitive dichotomy. This neuro-phenomenological account leans on phenomenological analyses and can straightforwardly explain recent neuroscience data. It emphasizes the dynamic, subjective, and piecemeal features of empathic experiences and unpicks the graded nature of empathy. *The graded empathy hypothesis* postulates that attending to others' expressions always facilitates empathy, but the parametric modulation in the levels of the empathic experience varies as a function of one's social interest (e.g., via intergroup or inter-personal cues) in the observed other. Drawing on multiple resources that integrate neuroscience with phenomenology, we describe the potential of this graded framework in an era of real-life experimentation. By wearing lenses of neuro-phenomenology, this original perspective can change the way empathy is considered.

## Philosophical Outlook on Empathy

### Phenomenological Definition

Empathy is a multifaceted phenomenon with several meanings depending on the context and discipline in which it is used. Contemporary debates in the philosophy of mind ascribe this term to our ability to grasp other subject-expressed mental states (1). This suggestion is in line with (2) concept of the German word “Einfühlung,” which was translated into English as empathy by (3). From a phenomenological perspective, empathy primarily amounts to direct perception of other subjects' mental states by attending to their facial expressions, gestures, and bodily patterns (4). (5) clarify this idea, noting: “I can attune to others' intentions and emotions on the basis of what I perceive of their behaviors and bodily expressions…. To the extent that I understand their intentions and emotions in this way, that just is what phenomenologists call empathy.” Nonetheless, for phenomenologists, empathy is not restricted to a basic sensory–motor attunement, but can extend to higher layers of interpersonal understanding (6, 7) that unfold as a function of the social situation at hand (8, 9); this will be detailed in the following sections.

### Inner Modeling (Simulation/Mentalizing)

Philosophers discuss the term empathy in the context of the question how we understand other minds. Contemporary debates in the philosophy of mind propose that emphatic understanding rests on either reflection (i.e., simulation) or introspection (i.e., mentalizing) [e.g., (10, 11)], both of which go beyond primary sensory–motor attunement (12). These approaches are based on the cartesian view that the mental is hidden and individualistic, and therefore the subject's emotions and attitudes are not accessible to other people.

Reflectionists suggest that empathy operates through a self-experience-based model. According to this hypothesis, which is known as “simulation theory,” attending to others' embodied behaviors generates a process of inner imitation. Consequently, the subject can understand others' attitudes and intentions from a first-person perspective without the need for mentalizing (9, 13). Proponents of introspectionism (i.e., mentalizing), which is known as “theory–theory” suggest that empathy unfolds at a higher level of intersubjectivity through a process of inference that is based on the acquisition of a “theory of mind” during the early phases of childhood. This ontogenetic transition occurs when children around the age of 4 years develop a capacity to infer others' beliefs and intentions [e.g., (14)]. Establishing a third-person point of view allows subjects to grasp others' motives through observation, and this facilitates empathic resonance (15).

### Beyond the Inner-Modeling Dichotomy

In contrast to both of these approaches, interaction theory, which is rooted in the phenomenological concept of direct perception (16), emphasizes the constitutive role of embodied engagements in fostering empathic understanding (17). Interactionists maintain that the socio-enactive character of humans' encounters (18) allows to immediately grasp others' embodied mental states without the need to employ self-experience-based model or reflect on their intentions and beliefs (9, 12). In other words, at a primary level, empathic understanding is manifested simply by attending to others' bodily behaviors within a social context. The focus on the role of environmental and intersubjective factors in driving interpersonal resonance downplays the dichotomy between the perceiver and the perceived (19). Interactionism shows that empathy is established through a dynamic process comprising a shared context, bodily expressions, and the impressions that these expressions trigger [(20), p. 33]. This approach emphasizes that in social encounters, we are not passively gathering information about other people. Rather, others' embodied behaviors are manifested and grasped in relation to the context of the encounter and the dynamics of our engagement (8, 21, 22). While “theory of mind” views in the philosophical literature emphasizes things that have an epistemic quality such as beliefs, intentions, and judgments, interactionists suggest that primary empathy can already unfold in young infants through attuning to rhythms and dynamics in dyadic interactions (23, 24). Interactionalism helps to unpick the graded nature of empathy by emphasizing that this early form of empathic resonance can extend in later ontogeny to include advanced types of interpersonal understanding (20).

## Neuroscientific Outlook on Empathy

### The Affective/Cognitive (i.e., Simulation/Mentalizing) Dichotomy

Along with the technological progress in neuroimaging, in the past couple of decades emerged the scientific research on the neural correlates of empathic responses. During the first decade of this millennium, evidence gradually accumulated to distinguish between affective (a.k.a., emotional, embodied simulation, or resonance) and cognitive (a.k.a., mentalization, theory of mind) empathy (25, 26). Accordingly, *affective empathy* (i.e., *simulation*) was ascribed to automatic processes reflecting vicarious pain and feelings; it was thought to emanate from sensorimotor and affective neural substrates: the sensorimotor cortex area, the anterior insula, and the anterior cingulate cortex. By contrast, *cognitive empathy* (i.e., *mentalizing*) was ascribed to higher-order processes reflecting vicarious mental states and understanding; it was proposed to emanate from higher-order cortices including the prefrontal cortex, temporo-parietal junction, and the superior temporal sulcus. Drawing parallels to other dichotomous models such as the lexical/phonological model of language (27, 28), the affective/cognitive model leaned on lesion studies (which are in themselves dichotomous) demonstrating direct mapping between specific neural systems and impairments in affective and cognitive empathy (25), and has allowed to explain various manifestations of empathy and its origins. For instance, different mental disorders like autism (29), schizophrenia (30) and psychopathy (31), or heritability variance (32). Further, studies on empathy development implemented the dichotomous framework to study the developmental trajectories of affective and cognitive empathy. For instance, it was claimed that the first emerges early in development (33), whereas the second has a more prolonged developmental course from childhood to adulthood (34, 35). Likewise, rudimentary neural networks are mostly in place by the end of infancy, whereas frontal areas reach maturity by young adulthood (36). This explains neurodevelopmental patterns of empathy: a complex change in the affective–cognitive empathy balance that matures with age both at the neuroanatomical-functional (37) and the neuro-rhythmicity (38–40) levels.

### Moving Beyond the Affective/Cognitive Dichotomy: Ecological Validity, Neural Mechanisms, and Phenomenological Considerations

Despite being paramount for the understanding of empathy, the dichotomous framework gradually revealed several limitations. First, it did not accommodate phylogenetic evidence pointing to the interconnected nature of the two components along evolution and across species (41). Second and perhaps more important to the current manuscript, dichotomous reports often leaned on simplistic designs and dualistic approaches that consolidated the validity of this dissociation. For instance, findings from numerous empirical experiments relied on simplistic artificial stimuli in tightly controlled lab contexts that convey distinct cerebral mapping patterns and that isolate one of the two components (42). Likewise, even in lesion studies (25), there was no direct dichotomous matching between the lesion and the behavioral outcome (43). Research on the multiple facets of empathy: neuroscience, development, heritability, and psychopathology—typically applied artificial and simplified experimental settings or models. In a way, methodology (e.g., questionnaires, coding schemes, stimuli) was developed and designed to pre-target the two components; hence, it was not surprising that findings straightforwardly matched the model. This parsimonious approach was crucial for neuroscientists to gain traction on the contribution of elemental socio-cognitive components (i.e., affective and cognitive) to the phenomenon of empathy (44). However, relying on overly simplified models (i.e., affective/cognitive dichotomy) did not allow drawing broader conclusions about empathy in more ecologically valid contexts, in particular, during interpersonal interaction (45) and intergroup contexts (46).

At the onset of the second decade of this millennium, a gradual emergence of naturalistic experimental settings began to establish in the cognitive and social neurosciences (47, 48), including in the neuroscience of empathy (44). This paradigm shift gradually conveyed the notion that this dichotomy is somewhat artificial and overestimates the dual distinction in live empathic encounters that are dynamic and interactive. As such, in 2015, a new lab paradigm was suggested to investigate the two systems in parallel (49). Further, the shift toward naturalistic experimentation showed a growing body of evidence that could no longer be accommodated by the dichotomous framework. For example, Goldstein et al. investigated brain-to-brain coupling during interpersonal empathic encounter and found that it was associated with the level of empathic accuracy of the empathizer (45). In another study, Levy et al. investigated the impact of intergroup representations on neural empathy and empathic behavior; the study found that empathy brain response was expressed by various rhythmic events occurring at different timings, and was amplified and synchronized as a function of intergroup representations and the emotions that they arose (46). These findings were hard to accommodate by the dichotomous model of empathy, and attempting to do so would miss important facets of the data. This is not surprising because in comparison to simplified and controlled experiments, experiments that involve naturalistic aspects of social life engage qualitatively different patterns of neural activity (50). Hence, to capture non-dualistic neural mechanisms, instead of relying on anatomical segregation, more advanced methods (e.g., multi-rhythmic temporal representations in MEG) should be employed (51, 52).

Beside the shift in methodology, phenomenological investigations, which by definition focus on lived experiences, also pointed out the need to move beyond dichotomy. For example, phenomenological studies of psychopathology suggest that anomalies of empathy in mental disorders do not necessarily rely on the affective–cognitive dichotomy, but rather unfold and amplify at both levels—often simultaneously (53). In autism, for instance, reduced capacity for attuning to affective cues (54) involves modification in the capacity to grasp others' mental states toward a shared context, and this amounts to difficulties in establishing gestalt perception of social scenes (55). The interplay between different aspects of empathy is also evident in other disorders: Schizophrenic patients show oscillations of self-other perspectives that diminish their ability to effectively follow others' embodied mental patterns and to discern their intentions (56, 57). In borderline personality disorder, and most likely in social anxiety disorder and posttraumatic stress disorder, the affective response to the bodily presence of others is altered, and this involves modifications in what are considered as “cognitive” aspects of empathy. Specifically, subjects with these types of disorders tend to overemphasize negative affective cues at the expense of other socio-affective stimuli (58), and this impacts interpersonal resonance and consequently the way the world appears to them (59).

Moreover, recent neuroimaging studies show that dichotomous modeling fails to accommodate empirical evidence that integrates lived experiences. A good example is the study by Grice-Jackson and colleagues on pain empathy (60, 61), which is basically elicited by observing others in painful situations (62). Typically, neuroscientists interpret pain empathy by implementing the dichotomous framework, thereby arguing that the vicarious perception of pain triggers simulation (63), while no mentalizing is elicited unless participants are explicitly instructed to take the targets' perspective (64, 65). By contrast, Grice-Jackson and colleagues examined empathy in the brain while integrating lived experiences (i.e., neuro-phenomenology) and found a graded phenomenon. The first group of participants (i.e., experiencers) reported no conscious experience of vicarious pain, the second group reported experiencing affect, and the third reported experiencing a sensorial and localized experience of pain while perceiving vicarious pain (61). This is a very good example of the difficulty in implementing the dichotomous affective/cognitive framework while relating to lived experiences of human beings. Noteworthy, a similar approach was recently conducted in two MEG studies while instead of investigating empathy, they addressed conscious perception (66, 67). In brief, while previous accounts claimed that conscious perception is dichotomous, that is, all-or-none [for a review, see (68)], phenomenal evidence pointed to a rather graded experience of conscious perception (69, 70). Similar the study of empathy (60, 61), by implementing a neuro-phenomenological approach, conscious perception was empirically demonstrated as a graded phenomenon (66, 67). *Altogether, inspired by a recent phenomenological outlook on levels of empathy that we describe in the following section, we contend that a new neuro-phenomenological framework is needed to accommodate the methodological paradigm shift and the necessity to integrate empirical measures with lived experiences*.

## The Phenomenological Account of Graded Empathy

The focus on the experiential features of empathy suggests that it is a multilevel process (7). Empathy can range from basic motor attunement to extended social understanding (12, 22), in accordance with the situation at hand and group factors (8, 9). (20) suggests that empathy consists of three levels of interpersonal understanding. In what follows, we draw on the phenomenological view on empathic understanding to develop a graded account, which emphasizes the crucial role of group contexts in shaping the levels of empathy.

### Primary Empathy

From a phenomenological perspective, the first layer of empathy is direct perception (71). Phenomenologists emphasize that in direct face-to-face encounters, we can immediately grasp other subjects' basic mental states by attending to their facial expressions and embodied patterns (16). This primary type of social understanding does not rely on imitation (i.e., simulation) or reflection (i.e., mentalizing). That is, primary empathy essentially amounts to a second person perspective process (72). A capacity for direct perception seems to be based on intersubjective predispositions such as fast detection and the prioritization of social stimuli that develop in the early stages of life (45, 73). These tendencies require mechanisms that allow the subject to quickly locate and discern others' embodied expressions (74–76).

A phenomenologically informed account of social understanding suggests that direct perception is enabled by the fact that the subject's mental world is not necessarily obscure from us (20). For phenomenologists, an expression is not a one-way process in which our inner world is on display; rather, our feelings are sometimes constituted and amplified by our embodied behaviors (77). In other words, bodily manifestations of emotions and intentions do not merely reflect an inner mental state, since the body also plays a constitutive role in shaping and communicating our experiences. Consequently, when attending to the expressions of others, we can actually see some of their mental operation (70). Furthermore, expressions have socio-communicative value. Expressions of emotions also unfold to provide others with information regarding the shared environment (21). This approach fits well with evolutionary theories that suggest that humans had evolved to share their emotions with others through facial expressions and embodied behaviors (78, 79).

Another feature that supports the capacity for direct perception is the participatory nature of social understanding (80). Phenomenological approaches to social cognition suggest that empathic resonance is attained through a dynamic process, which involves two (or more) lived bodies (9, 18, 81). By virtue of the unique phenomenal structure of intersubjectivity, social perception is phenomenologically and ontologically distinct, to begin with (82) and (83). When encountering other subjects, we immediately recognize a differentiated subjectivity (6). This occurs because the other person's body, like my own, is not experienced as an inanimate object, but rather as a field of their lived experiences. (22) clarifies this idea noting that the other's body is “present to me as a field of expression for his subjective experience” (p. 163). This allows the subject to quickly and effectively gain other subjects' perspectives by locating and following their embodied patterns and facial expressions. (22) analysis also shows that social understanding is not necessarily a one-way street. The perception of others' mental operation is intensified by the socio-dynamic nature of the encounter. In everyday life, the social background of our interpersonal engagements typically facilitates a two-step process (12). First, the other's expressive behavior, such as expressions of anger, triggers bodily arousal, which precedes other types of operation (24). Then, the observer bodily responses drive an interactive set of feedbacks, comprising expressions and impressions (19, 20, 84). This socio-affective cycle allows a dynamic space (85), in which empathic understanding derives from the subjective framework of the encounter.

These considerations suggest that empathy does not necessitate at the primary level inner-imitation or reflection [e.g., (9, 11)]. Empathic understanding is enabled, primarily by the fact that it is directed at a differentiated subjectivity. As (9) explains: “To have a feeling of oneself and to know that another has it are two fundamentally different things. The first is not conditioned in the second, nor the second in the first” (1979, p. 25)^1^. Investigations into the phenomenal structure of humans' interpersonal encounters show that attention to others' embodied expressions always triggers minimal empathy. This idea is illustrated in the “*boulevard example*” [(21), p. 389]: Imagine a situation where I walk down the boulevard and a person approaches me from the opposite direction. While we pass, I notice her/his slightly bent posture and part of her/his reddish sad face. Attention to the expressive behavior in these situations triggers a minimal type of empathy in the sense that I pre-reflectively grasp the other's sadness (16), regardless of any imitation, reflection, or social operation (87, 88).

This example demonstrates that primary empathy requires nothing more than detecting and following others' expressions; this is precisely what the first level of empathy amounts to. This view gains support from empirical studies that suggest that empathic understanding is established and regulated at early developmental stages through sensory–motor attunement to others' embodied patterns (45, 89). *Hence, the primary type of empathic response that arises in social encounters is immediate, does not rely on simulation or mentalizing, and is unconditioned by any kind of social operation*.

### Secondary Empathy

Empathy, however, can go beyond the primary level; this occurs when direct perception opens the door to deepen empathic understanding. Typically, the amplified forms of empathy are driven by communal predispositions (18, 81). In everyday situations, my emotional attachment and commitment to the people I encounter influences my interest in their expressions and this leads to heightened types of empathy (9, 90). Specifically, the incorporation of broadened affective ties^2^ into social perception constitutes extended empathic layers, these layers comprise: (A) envisioning how the world appears from the other person's perspective (i.e., secondary empathy), and (B) the other's stance toward me during the emphatic encounter, which is typical of conditions of group cohesion (i.e., tertiary empathy) (7, 20).

Phenomenologists emphasize that regardless of the level of empathy that attention to others triggers, empathic experiences always stem from the self-other distinction (19). That is to say, empathy is directed at other subjects' experiential world and recognizes their differentiated embodied selfhood. As [(88), p. 92] puts it, “The phenomenologists would consequently reject the view that imitation, emotional contagion or mimicry should be the paradigm of empathy.” This approach does not necessarily rule out the possibility that empathic understanding extends by my interest in the other subject (91). Indeed, it is precisely because empathic processes are other-directed that empathy can increase by virtue of the nature of our we-relationship (22); the more I am emotionally attached to the person I attend to, the more I am interested in their mental states, and correspondingly empathy amplifies [for the impact of emotions on social cognition see in (92)].

At the phenomenal level, variations to the empathic process are induced by a social factor (i.e., commitment or attachment). In the previous *boulevard example*, due to my social interest in the person walking past me, I sometimes also take her/his position toward the situation through an imaginary process or even go deeper to reflect on her/his motives. Both cases cannot merely rest on imitating the others' emotional state as proponents of the simulation theory claim (9, 13) ^3^.

The first experiential step toward a fully amplified empathic response that emotional commitment induces go through taking the other's perspective. This entails an imaginative operation, which manifests itself as an *as if* scenario (20). By virtue of this operation, I experience the other person's sadness, also by taking their stance. Secondary empathy often unfolds in situations where I have more interest in the attended other (7). [(20), p. 38] suggests that this materializes in cases of disturbances, such as a misunderstanding or irritation. Yet, it seems that the second level of empathy is generated primarily by the fact that I am emotionally committed or attached to the attended other, and therefore, I am driven to take their position by employing an imaginary model.

Usually, to explicate others' experiences in a way that includes taking their perspective, i.e., *as if* I were in their shoes, requires some degree of emotional attachment/commitment. This intersubjective component allows the incorporation of an implicit socio-attentional process (53), with an explicit operation that is based on the capacity to grasp others' differentiated perspective (93). In everyday situations, including in cases of disturbances, the amplification of the empathic process is intimately related to the nature of our relationship. Social ties often trigger an *as if* imaginary process, which increases empathy. For example, when the expressions of the person I encounter suggest that she/he is irritated, the expressive behavior and the social context allows primary empathy (12). Nonetheless, in order for me to experience how I would feel and react if I were in her/his place requires an additional empathic step. This secondary intersubjective phase necessitates that I have an interest in the other subject, which transcends the temporal encounter. Social interests that amplify empathic underspending are typically constituted by communal concerns. These concerns may involve manifold social relationships (9). Aroused by a pre-reflective induced communal-based interest, subjects are more prone in some situations to employ a socio-imaginary operation, which is incorporated into the empathic process. This secondary layer extends, as we show next, in cases of increased social attachment. Hence, *secondary empathy is driven by a communal-based interest and requires the process of perspective taking*.

### Tertiary Empathy

**Figure 1:**
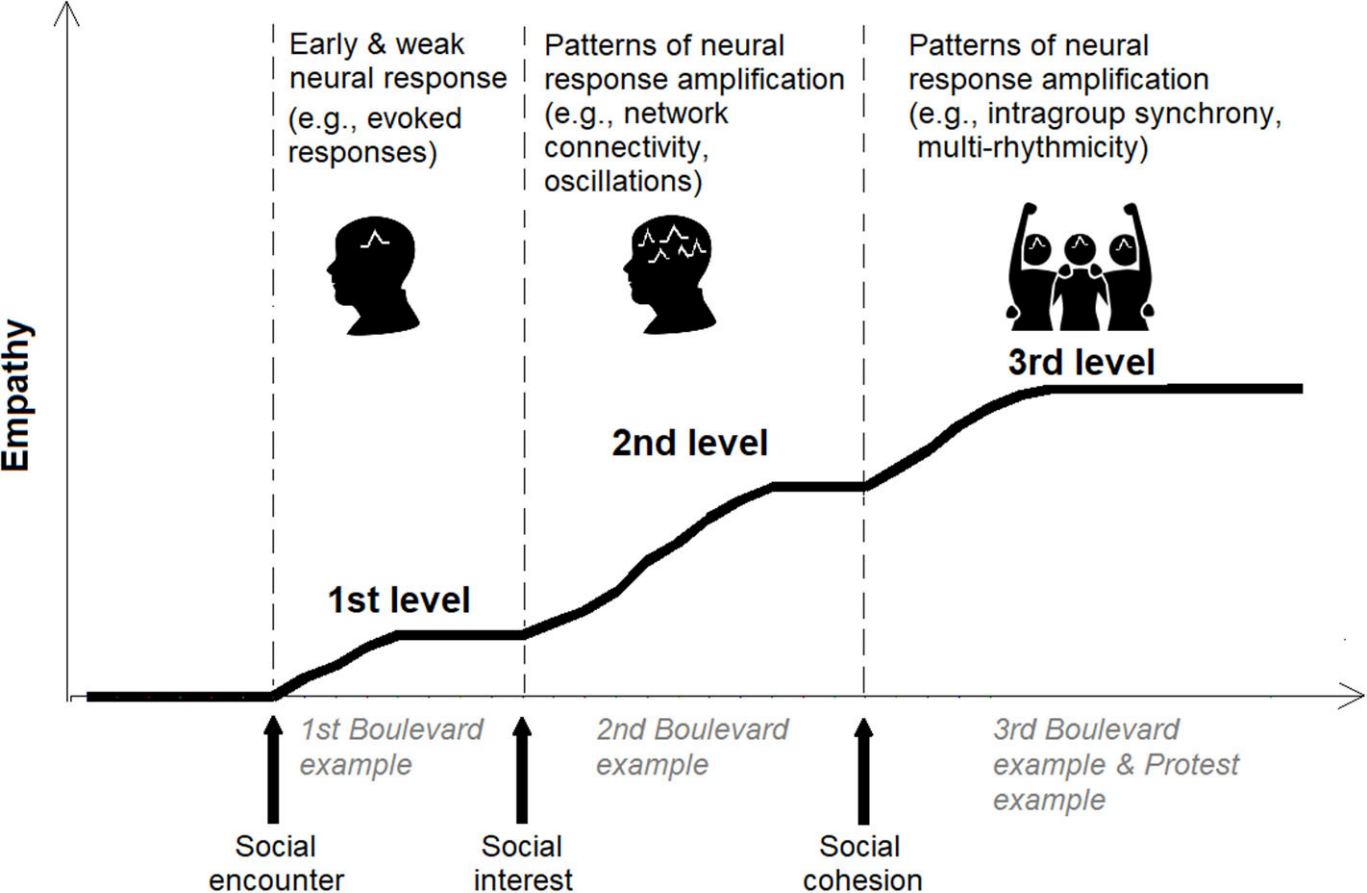
Illustration of the neuro-phenomenological graded empathy framework. The first level of empathy is elicited by the empathic encounter (evoking a minimal neural response). If there is social interest in the target, enhanced neural activity is elicited (involving heightened complexity in terms of neural rhythms and sources), while during intense social cohesion (the target is perceived in group contexts), the neural response is further amplified while conveying patterns of neural cohesion. Real-life examples (i.e., the Boulevard and the Protest examples) are provided in The Phenomenological Account of Graded Empathy section of the manuscript to further illustrate the three levels.

In comparison to the first and second levels that relate to individual targets, the third empathic level is driven by group factors (i.e., intergroup relations) (c.f., Figure 1). Phenomenologists suggest that the third level of empathy consists of an experiential structure in which I perceive myself from the other's perspective as she/he perceives me attending to her/his expressions (20). In these cases, the nature of our relationship drives interpersonal understanding that goes beyond an *as if* scenario. (7) maintains that at this phase of empathy, the other's expression is given to me as an intentional object that I can reflect upon (91). We argue that tertiary empathy unfolds in two types of encounters that are colored by intense group interest: those that do not necessitate mutual emphatic awareness, and those that rely on it. The first unfolds in situations that involve a strong sense of social cohesion (94), such as a case in which one observes a member of her/his group in conflict situations (even if she/he is not aware of the other's attention to her/his expressive behavior). In these settings, a fused perspective provoked by increased emotional commitment is fueled by the scene's circumstances and manifests itself as tertiary empathy. That is, the strong sense of identification with the other person incorporated with my attention to the scene triggers an amplified empathic process. This concept is nicely illustrated by what we label as “*the protest example*”: a situation where I participate in a protest against the government's corruption. At some point, I notice that a member of the group is dragged by police officers. Even if the other person is not directly aware of my attention to the scene (or even of my presence), my empathic experience will typically go beyond placing myself in his/her shoes to include motives and beliefs that led to the situation.

The second type of tertiary empathy is based on increased sense of social interest, which is broadened through mutual awareness. As we suggest in the previous subsection, extended types of empathy are often triggered by social ties. Manifestations of tertiary empathy require that this critical factor significantly intensifies. Reframing tertiary empathy in the *boulevard example*, let us assume that it turns out that I am attached to the sad person approaching me in the boulevard by virtue of increased group interests (e.g., family, friendship, or other close ingroup ties). While we pass, she/he observes me attending to her/his sadness. Typically, this situation stimulates an imaginary operation, where I take the other's perspective. However, it also can drive deeper emphatic responses. This occurs as a result of mutual awareness, which is amplified by group-based-factors^4^. Consequently, this emphatic step will address motives and events (both actual and fictional) that are beyond my direct experiential reach (7). Hence, *tertiary empathy typically arises in the context of heightened social cohesion*.

## Graded Empathy Through The Lenses of Neuro-Phenomenology

In the previous section, we formulated that social bonding increases empathic responses and shed light on the graded nature of empathy, thereby undermining the affective/cognitive dichotomy in certain contexts. The constitutive role of group factors in determining the levels of empathic understanding, which is indicated by phenomenological analyses of social encounters (9, 90), shows that the amplification of empathy involves increased group ties with broadened cognitive operations. Altogether, our phenomenal typology suggests that in its fully amplified form, empathy involves three steps that are spontaneously activated during the encounter. As was highlighted in the “*boulevard example*,” the more I am emotionally engaged (i.e., via interpersonal or intergroup representations) with the target of empathy, the more empathy is amplified. In the present section, following in the footsteps of Francisco (95) concept of neuro-phenomenology, we integrate this phenomenological account with neuroscientific findings. Varela coined the term to describe a research area “in which lived experience and its natural biological basis are linked by mutual constraints provided by their respective descriptions” [(95), p. 112]. The phenomenological outlook described in the previous section emphasizes the lived encounters, feedback, dynamic, and graded parametric aspects in empathic encounters, and therefore, a graded framework better accommodates real-life experiences compared to a dichotomous view.

In the Moving Beyond the Affective/Cognitive Dichotomy: Ecological Validity, Neural Mechanisms, and Phenomenological Considerations section, we detailed the limitations of the affective/cognitive approach in accommodating data that describe intergroup conditions, naturalistic designs, and phenomenological approaches. We now turn to detail how neural mechanisms in these recent data can be explained according to the graded framework. As outlined above, primary empathy is a basic intrinsic perceptual process unconditioned by social operation, and this can be explained by the almost immediate (i.e., ~100-ms poststimulus onset) neural response to empathy-evoking targets (96). This response is amplified as a function of social factors, as can be evidenced in numerous studies investigating the neural empathic response (39, 40, 46, 65, 97–103). Yet, these findings are also explained by the dichotomous framework of empathy, for instance, by explaining differences in neural substrates (i.e., lower vs. higher-order cortices) and chronometry (i.e., early vs. late response) as a function of the affective and cognitive components of empathy, respectively. However, in contrast to this dichotomous model, the graded framework straightforwardly accommodates recent empathy neuroimaging experiments that integrate phenomenological reports (60, 61), as well as experiments targeting complex interpersonal and intergroup contexts and employing naturalistic experimental settings.

For instance, the ingroup representations amplify empathy to the tertiary level by triggering a strong sense of social cohesion and emotional attachment between the empathizer and the target. From a biological perspective, our brain has an innate and instinctual propensity to distinguish between friend and foe (104, 105), resulting in amplified empathy for kin (i.e., the ingroup) compared to non-kin (i.e., outgroups) (106). In recent years, there is a growing body of neuroscientific research on intergroup empathy, so this topic can provide ample empiric evidence for the amplification of empathy, particularly toward the tertiary level. Early neuroimaging studies that examined empathy in intergroup contexts showed that the neural empathic response is difficult to interpret in the affective/cognitive terminology particularly while using naturalistic stimuli and real-life design, but can be explained via the graded framework. For example, Hein et al. showed that the more one's empathy toward ingroup targets was amplified, the more one was willing to engage in costly helping toward the ingroup target (107). In a more recent similar study, MEG was used and this enabled to track over time the amplification of various neural empathic mechanisms toward ingroup and outgroup targets (46) (see Box 1). In another study that emphasized ecological validity, brain-to-brain coupling was measured during real-life interpersonal empathic encounters (45); as in the intergroup study (46), the encounter involved strong social cohesion, but this time due to romantic partnership. The authors found that interbrain coupling in the alpha-band reduces partners' pain and is amplified by empathic accuracy. Another study that investigated interbrain coupling during mother–child encounter, while using naturalistic and, at the same time, controlled experimental settings (108); once again, the social cohesion factor was enhanced due to the strong mother–child bond. The authors found interbrain coupling and activation in the gamma-band, conveying empathy being amplified by cohesion (i.e., reciprocity and synchrony). Altogether, we illustrate in Figure 1 the graded empathy framework and the suggested neural mechanisms that convey the amplification as a function of social factors.

Box 1 **Empirical illustration of the *Graded Empathy Hypothesis*.**

In our recent study, we investigated empathy among 80 adolescent high-school students. The adolescents lay down during an MEG neuroimaging session, while facing a screen projecting stimuli of hands or feet in painful (vs. non-painful as control) situations, thereby probing participants' empathy brain response to others' pain in general, or as a function of targets' group membership (Figure 1). Following the MEG session, participants interacted with each other and we monitored their social behavior (46). Findings revealed that adolescents' brain response to the pain of others emerged early (<200 ms) after stimuli onset by a neural mechanism of alpha-band suppression; this early neural response remained unchanged as a function of group context. This early, yet weak response of the brain to vicarious pain matches the assumption of a first layer of empathy (i.e., primary): (a) elicited almost immediately following the empathic encounter, and (b) unconditioned by any social operation. Further to the early neural response, a later (>500 ms) and more robust response emerged as a second neural mechanism (i.e., alpha-band rebound), and only toward ingroup targets. Importantly, the latter mechanism was amplified as a function of intergroup interest (i.e., hostility). Finally, another level of intergroup interest (i.e., lack of empathy) strongly amplified a third mechanism—group neural synchrony. These two latter neural mechanisms corroborate the phenomenological assumption that social interest, and in particular social cohesion, act as strong amplifiers of the empathic response.

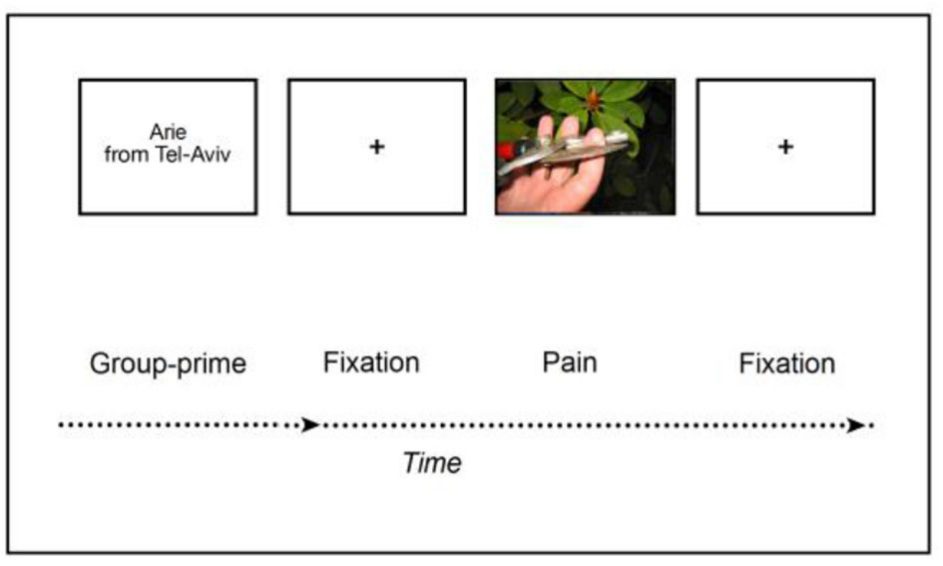

Finally, the idea that empathy operates in a graded manner, pending on social circumstances, might also benefit the design of prevention program for individuals with difficulties in empathic understanding, in that it suggests that it could be useful for treatment models to pay more attention to group behaviors (such as collective intentionality) rather than solely focusing on mentalizing capacities. Several strategies have been proposed to promote empathy, including literary fiction (109), virtual reality (110), or intergroup dialog (111, 112). The success or failure of these interventions may additionally address a central question: whether empathy is innate or, alternatively, whether it can be learned and fostered. In the context of the hypothesis raised in the current manuscript, we emphasize the importance of integrating neuroscience and phenomenology into empathy-building intervention studies. We will end by raising several outstanding questions regarding the graded framework for empathy. Are there specific neural signatures for each of the three levels? What is the nature of the interaction between these levels from a neuronal perspective? Does the framework apply to other social phenomena? What experimental designs can further advance the mapping between phenomenology and empathy neuroscience? Would the graded framework highlight specific neural patterns in psychopathology, development, and heritability? Would future neuroscience findings propose additional levels to the model? More empiric research is needed to address these questions and build upon this framework in the future. The answers to these questions can also be informative for further understanding the operation of empathy in daily circumstances.

To conclude, by providing this neuro-phenomenological framework, we address the recent call (113) for social neuroscience to connect basic neurocognitive processes to a broader array of intergroup contexts and their real-world outcomes. Our model's novelty lies in the fact that (a) it explains why in real-life situations it is insufficient to solely rely on the cognitive/emotional dichotomy to describe the experience of empathy, in (b) suggesting an original conceptualization explaining the amplification of empathic responses, which is something that the prevailing accounts, as yet, have failed to achieve, and finally, (c) it distinguishes empathic experiences as a function of their social/group context; this stands out in comparison to the dichotomous account that rather relies on simulation/mentalizing or bottom–up/top–down considerations. Nevertheless, the model proposed here does not “negate” the cognitive/emotional framework; instead of dichotomizing, the novel model offers a dynamic and graded outlook that can change the way empathy is considered, particularly in intergroup contexts and while implementing real-world experimentation.

## Data Availability Statement

The original contributions presented in the study are included in the article/supplementary material, further inquiries can be directed to the corresponding author/s.

## Author Contributions

All authors listed have made a substantial, direct and intellectual contribution to the work, and approved it for publication.

## Conflict of Interest

The authors declare that the research was conducted in the absence of any commercial or financial relationships that could be construed as a potential conflict of interest.

## Footnotes

^1^Gurwitsch, one of the key figures in the phenomenological tradition, offers a conceptual understanding of the subjective structure of empathy. Gurwitsch philosophical definition of the experience of others' basic mental states is aligned with the scientific research on subjects with congenital insensitivity to pain (CIP) that suggest that although CIP patients cannot refer to their own experience of pain, they show normal responses to observed pain (86).

^2^In the sense that they exceed the basic dyadic types of inter-affectivity (45).

^3^For simulation theory, subjects gain access to others' minds by running an inner simulation of their behavior (as-if scenario), which is then attributed back to the encountered other (20).

^4^I.e., I perceive the other's awareness of my perception of her/his expression.

## References

[1] Bizzari V Dambha-Miller H Laughaey WF Carvalho C. Defining therapeutic empathy: the philosopher's view. J R Soc Med. (2019) 112:91–5. 10.1177/0141076819831869 30868927 PMC6423527

[2] Lipps T Einfühlung, innere nachahmung und organenempfindung. Arch Gesamte Psychol. (1903) 2–3:185–204

[3] Titchener EB Lectures on the Experimental Psychology of Thought-Processes. New York, NY: Macmillan (1909).

[4] Zahavi D Empathy and mirroring: husserl and gallese. Phaenomenologica. (2012) 201:217–54.

[5] Gallagher S Gallagher J Acting oneself as another: an actor's empathy for her character. Topoi. (2019). 10.1007/s11245-018-9624-7

[6] Husserl E Zur Phänomenologie der Intersubjektivität I, Husseriana 13. Den Haag: Martinus Nijhoff (1973).

[7] Stein E On the Problem of Empathy. Washington, DC: ICS Publishers (1989).

[8] Fuchs T The phenomenology and development of social perspectives. Phenomenol Cogn Sci. (2013) 12:655–83. 10.1007/s11097-012-9267-x

[9] Gurwitch A Human Encounters in the Social World. Pittsburg, PA: Duquesne University Press (1979).

[10] Goldman A Simulating Minds: The Philosophy, Psychology and Neuroscience of Mindreading. Oxford: Oxford University Press (2006).

[11] Gopnik A Wellman HM Why the child's theory of mind really is a theory. Mind Lang. (1992) 7:145–71.

[12] Gallagher S Neurons, neonates and narrative: From embodied resonance to empathic understanding. In: Foolen A Lüdtke U Zlatev J Racine T, editors. Moving Ourselves, Moving Others. Amsterdam: John Benjamins (2012). p. 167–96.

[13] Gallese V Goldman A. Mirror neurons and the simulation theory of mind-reading. Trends Cogn Sci. (1998) 12:493–501. 10.1016/s1364-6613(98)01262-5 21227300

[14] Baron-Cohen S Mindblindness: An Essay on Autism and Theory of Mind. Cambridge, MA: MIT Press (1995).

[15] Carruthers P. Mindreading underlies metacognition. Behav Brain Sci. (2009) 32:164–76. 10.1017/S0140525X09000831 19386144

[16] Krueger J Seeing mind in action. Phenomenol Cogn Sci. (2012) 11:149–73. 10.1007/s11097-011-9226-y

[17] Gallagher S How the Body Shapes the Mind. New York, NY: Oxford University Press (2005).

[18] Fuchs T De Jaegher H Enactive intersubjectivity: participatory sense-making and mutual incorporation. Phenomenol Cogn Sci. (2009) 8:465–86. 10.1007/s11097-009-9136-4

[19] Zahavi D You, me and we: the sharing of emotional experiences. J Conscious Stud. (2015) 22:84–101.

[20] Fuchs T Levels of empathy: Primary, extended, and reiterated empathy. In: Lux V Weigel S, editors. Empathy. Epistemic Problems and Cultural-Historical Perspectives of a Cross-Disciplinary Concept (p). Basingstoke: Palgrave Macmillan (2017). p. 27–47.

[21] Bader O. Attending to emotions is sharing of emotions-a multidisciplinary perspective to social attention and emotional sharing. Comment on Zahavi and Rochat (2015). Conscious Cogn. (2016) 42:382–95. 10.1016/j.concog.2016.04.012 27152930

[22] Schutz A The Phenomenology of the Social World. G. Walsh (trans.), Evanston, IL: Northwestern University Press (1967).

[23] Reddy V A gaze at grips with me. In: Seemann A, editor. Joint Attention: New Developments in Philosophy, Psychology, and Neuroscience. Cambridge, Mass: MIT Press (2012). p. 137–57

[24] Stern DN. The Interpersonal World of the Infant. New York, NY: Basic Books (1985).

[25] Shamay-Tsoory SG Aharon-Peretz J Perry D. Two systems for empathy: a double dissociation between emotional and cognitive empathy in inferior frontal gyrus versus ventromedial prefrontal lesions. Brain. (2009) 132:617–27. 10.1093/brain/awn279 18971202

[26] Bernhardt BC Singer T. The neural basis of empathy. Annu Rev Neurosci. (2012) 35:1–23. 10.1146/annurev-neuro-062111-150536 22715878

[27] Coltheart M Rastle K Perry C Langdon R Ziegler J. DRC: a dual route cascaded model of visual word recognition and reading aloud. Psychol Rev. (2001) 108:204–56. 10.1037/0033-295X.108.1.204 11212628

[28] Levy J Pernet C Treserras S Boulanouar K Aubry F Démonet JF. Testing for the dual-route cascade reading model in the brain: an fMRI effective connectivity account of an efficient reading style. PLoS ONE. (2009) 4:e6675. 10.1371/journal.pone.0006675 19688099 PMC2724737

[29] Smith A The empathy imbalance hypothesis of autism: a theoretical approach to cognitive and emotional empathy in autistic development. Psychol Rec. (2009) 59:489–510. 10.1007/BF03395675

[30] Bonfils KA Lysaker PH Minor KS Salyers MP. Affective empathy in schizophrenia: a meta-analysis. Schizophr Res. (2016) 175:109–17. 10.1016/j.schres.2016.03.037 27094715

[31] Blair RJR. Responding to the emotions of others: dissociating forms of empathy through the study of typical and psychiatric populations. Conscious Cogn. (2005) 14:698–718. 10.1016/j.concog.2005.06.004 16157488

[32] Abramson L Uzefovsky F Toccaceli V Knafo-Noam A. The genetic and environmental origins of emotional and cognitive empathy: review and meta-analyses of twin studies. Neurosci Biobehav Rev. (2020) 114:113–33. 10.1016/j.neubiorev.2020.03.023 32353470

[33] Tousignant B Eugène F Jackson PL. A developmental perspective on the neural bases of human empathy. Infant Behav Dev. (2017) 48:5–12. 10.1016/j.infbeh.2015.11.006 26995647

[34] Eisenberg N Cumberland A Guthrie IK Murphy BC Shepard SA. Age changes in prosocial responding and moral reasoning in adolescence and early adulthood. J Res Adolesc. (2005) 15:235–60. 10.1111/j.1532-7795.2005.00095.x 20592955 PMC2893741

[35] Decety J Michalska KJ. Neurodevelopmental changes in the circuits underlying empathy and sympathy from childhood to adulthood. Dev Sci. (2010) 13:886–99. 10.1111/j.1467-7687.2009.00940.x 20977559

[36] Casey BJ Tottenham N Liston C Durston S Imaging the developing brain: what have we learned about cognitive development? Trends Cogn Sci. (2005) 9:104–10. 10.1016/j.tics.2005.01.011 15737818

[37] Decety J Michalska KJ Kinzler KD. The contribution of emotion and cognition to moral sensitivity: a neurodevelopmental study. Cereb Cortex. (2012) 22:209–20. 10.1093/cercor/bhr111 21616985

[38] Levy J Goldstein A Pratt M Feldman R. Maturation of pain empathy from child to adult shifts from single to multiple neural rhythms to support interoceptive representations. Sci Rep. (2018) 8:1810. 10.1038/s41598-018-19810-3 29379042 PMC5788915

[39] Levy J Goldstein A Feldman R. The neural development of empathy is sensitive to caregiving and early trauma. Nat. Commun. (2019) 10:1905. 10.1038/s41467-019-09927-y 31015471 PMC6478745

[40] Levy J Yirmiya K Goldstein A Feldman R. The neural basis of empathy and empathic behavior in the context of chronic trauma. Front Psychiatry. (2019) 10:562. 10.3389/fpsyt.2019.00562 31474883 PMC6706815

[41] de Waal FBM Preston SD. Mammalian empathy: behavioural manifestations and neural basis. Nat Rev Neurosci. (2017) 18:498–509. 10.1038/nrn.2017.72 28655877

[42] Keysers C Gazzola V. Integrating simulation and theory of mind: from self to social cognition. Trends Cogn Sci. (2007) 11:194–6. 10.1016/j.tics.2007.02.002 17344090

[43] Perry A Saunders SN Stiso J Dewar C Lubell J Meling TR. Effects of prefrontal cortex damage on emotion understanding: EEG and behavioural evidence. Brain. (2017) 140:1086–99. 10.1093/brain/awx031 28334943 PMC6075458

[44] Zaki J Ochsner K. The neuroscience of empathy: progress, pitfalls and promise. Nat Neurosci. (2012) 15:675–80. 10.1038/nn.3085 22504346

[45] Goldstein P Weissman-Fogel I Dumas G Shamay-Tsoory SG. Brain-to-brain coupling during handholding is associated with pain reduction. Proc Natl Acad Sci USA. (2018) 115:E2528–37. 10.1073/pnas.1703643115 29483250 PMC5856497

[46] Levy J Goldstein A Influs M Masalha S Zagoory-Sharon O Feldman R. Adolescents growing up amidst intractable conflict attenuate brain response to pain of outgroup. Proc Natl Acad Sci USA. (2016) 113:13696–701. 10.1073/pnas.1612903113 27849588 PMC5137744

[47] Hari R Henriksson L Malinen S Parkkonen L. Centrality of social interaction in human brain function. Neuron. (2015) 88:181–93. 10.1016/j.neuron.2015.09.022 26447580

[48] Sonkusare S Breakspear M Guo C. Naturalistic stimuli in neuroscience: critically acclaimed. Trends Cogn Sci. (2019) 23:699–714. 10.1016/j.tics.2019.05.004 31257145

[49] Kanske P Böckler A Trautwein FM Singer T. Dissecting the social brain: Introducing the empatom to reveal distinct neural networks and brain-behavior relations for empathy and theory of mind. Neuroimage. (2015) 122:6–19. 10.1016/j.neuroimage.2015.07.082 26254589

[50] Shamay-tsoory SG Mendelsohn A. Real-Life neuroscience : an ecological approach to brain and behavior research. Perspect Psychol Sci. (2019) 14:841–59. 10.1177/1745691619856350 31408614

[51] Gross J. Magnetoencephalography in cognitive neuroscience: a primer. Neuron. (2019) 104:189–204. 10.1016/j.neuron.2019.07.001 31647893

[52] Levy J Lankinen K Hakonen M Feldman R. The integration of social and neural synchrony: a case for ecologically valid research using MEG neuroimaging. Soc Cogn Affect Neurosci. (2020) 1–10. 10.1093/scan/nsaa061 32382751 PMC7812634

[53] Bader O. Alterations of social attention in mental disorders: phenomenology, scope, and future directions for research. Conscious Cogn. (2020) 79:102884. 10.1016/j.concog.2020.102884 32032824

[54] Fuchs T Pathologies of intersubjectivity in autism and schizophrenia. J Consc Stud. (2015) 22:191–214.

[55] Klin A Jones W Schultz R Volkmar F The enactive mind, or from actions to cognition: lessons from autism. Philos Trans R Soc Lond B Biol Sci. (2003) 358:345–60. 10.1098/rstb.2002.1202 12639332 PMC1693114

[56] Fuchs T Phenomenology and psychopathology. In: Gallagher S Schmicking D, editors. Handbook of Phenomenology and the Cognitive Sciences. Dordrecht: Springer (2010). p. 547–3.

[57] Kring AM Elis O. Emotion deficits in people with schizophrenia. Ann Rev Clin Psychol. (2013) 9:409–33. 10.1146/annurev-clinpsy-050212-185538 23245340

[58] Herpertz SC Bertsch K The social-cognitive basis of personality disorders. Curr Opin Psychiatry. (2014) 27:73–77. 10.1097/YCO.0000000000000026 24270477

[59] Bader O The human extended socio-attentional field and its impairment in borderline personality disorder and in social anxiety disorder. Phenomenol Cogn Sci. (2019) 19:169–89. 10.1007/s11097-019-09621-w

[60] Grice-Jackson T Critchley HD Banissy MJ Ward J. Consciously feeling the pain of others reflects atypical functional connectivity between the pain matrix and frontal-parietal regions. Front Hum Neurosci. (2017) 11:507. 10.3389/fnhum.2017.00507 29104537 PMC5655021

[61] Grice-Jackson T Critchley HD Banissy MJ Ward J. Common and distinct neural mechanisms associated with the conscious experience of vicarious pain. Cortex. (2017) 94:152–63. 10.1016/j.cortex.2017.06.015 28759805

[62] Osborn J Derbyshire SWG. *Pain* sensation evoked by observing injury in others. Pain. (2010) 148:268–74. 10.1016/j.pain.2009.11.007 20005042

[63] Lamm C Decety J Singer T. Meta-analytic evidence for common and distinct neural networks associated with directly experienced pain and empathy for pain. Neuroimage. (2011) 54:2492–502. 10.1016/j.neuroimage.2010.10.014 20946964

[64] Lamm C Batson CD Decety J. The neural substrate of human empathy: effects of perspective-taking and cognitive appraisal. J Cogn Neurosci. (2007) 19:42–58. 10.1162/jocn.2007.19.1.42 17214562

[65] Fan Y Han S. Temporal dynamic of neural mechanisms involved in empathy for pain: an event-related brain potential study. Neuropsychologia. (2008) 46:160–73. 10.1016/j.neuropsychologia.2007.07.023 17825852

[66] Andersen LM Pedersen MN Sandberg K Overgaard M. Occipital MEG activity in the early time range. (<300 ms) predicts graded changes in perceptual consciousness. Cereb Cortex. (2016) 26:2677–88. 10.1093/cercor/bhv108 26009612

[67] Levy J Vidal JR Fries P Démonet JF Goldstein A. Selective neural synchrony suppression as a forward gatekeeper to piecemeal conscious perception. Cereb Cortex. (2016) 26:3010–22. 10.1093/cercor/bhv114 26045565

[68] Dehaene S Changeux J-P. Experimental and theoretical approaches to conscious processing. Neuron. (2011) 70:200–27. 10.1016/j.neuron.2011.03.018 21521609

[69] Overgaard M Rote J Mouridsen K Ramsøy TZ Is conscious perception gradual or dichotomous? A comparison of report methodologies during a visual task. Consc Cogn. (2006) 15:700–8. 10.1016/j.concog.2006.04.002 16725347

[70] Overgaard M Mogensen J. Visual perception from the perspective of a representational, non-reductionistic, level-dependent account of perception and conscious awareness. Philos Trans R Soc B Biol Sci. (2014) 369:20130209. 10.1098/rstb.2013.0209 24639581 PMC3965164

[71] Zahavi D Rochat P. Empathy = sharing: perspectives from phenomenology and developmental psychology. Conscious Cogn. (2015) 36:543–53. 10.1016/j.concog.2015.05.008 26070850

[72] Schilbach L Timmermans B Reddy V Costall A Bente G Schlicht T. Toward a second-person neuroscience. Behav Brain Sci. (2013) 36:393–414. 10.1017/S0140525X12000660 23883742

[73] Farroni T Csibra G Simion F Johnson MH. Eye contact delectation in humans from birth. Proc Natl Acad Sci. (2002) 99:9602–5. 10.1073/pnas.152159999 12082186 PMC123187

[74] Crouzet SM Kirchner H Thorpe SJ. Fast saccades toward faces: face detection in just100 ms. J Vision. (2010) 10:1–17. 10.1167/10.4.16 20465335

[75] Morand SM Harvey M Grosbras MH Parieto-occipital cortex shows early target selection to faces in a reflexive orienting task. Cereb Cortex. (2012) 24:898–907. 10.1093/cercor/bhs368 23183710

[76] Pitcher D Goldhaber T Duchaine B Walsh V Kanwisher N Two critical and functionally distinct stages of face and body perception. J Neurosci. (2012) 32:15877–85. 10.1523/JNEUROSCI.2624-12.2012 23136426 PMC3752141

[77] Merleau-Ponty M Signs. Evanston, IL: Northwestern University Press (1964).

[78] Darwin C The Expression of Emotions in Man and Animals. Oxford: Oxford University Press (1998).

[79] Hrdy S Mothers and Others: The Evolutionary Origins of Mutual Understanding. Cambridge, MA: Harvard University Press (2009).

[80] De Jaegher H Di Paolo EA. Participatory sense-making: an enactive approach to social cognition. Phenomenol Cogn Sci. (2007) 6:485–507. 10.1007/s11097-007-9076-922701412

[81] Gurwitch A Phenomenology and Psychology. Evanston, IL: Northwestern University Press (1966).

[82] Sartre JP Being Nothingness. Barnes HE (Trans.). London: Routledge (2003).

[83] Reddy V Engaging minds in the first year: the developing awareness of attention intention. In: Bremner G, editor. Handbook of Infant Development, 2nd ed Oxford: Wiley-Blackwell (2010). p. 365–93.

[84] Overgaard S Krueger J. Social perception “Spectator Theories” of other minds. Commentary on Schilbach et al. Behav Brain Sci. (2013) 36.4:434–5. 10.1017/S0140525X12002014 23883763

[85] Krueger J Ontogenesis of the socially extended mind. Cogn Syst Res. (2013) 25–26:40–6. 10.1016/j.cogsys.2013.03.001

[86] Danziger N Faillenot I Peyron R. Can we share a pain we never felt? Neural correlates of empathy in patients with congenital insensitivity to pain. Neuron. (2009) 61:203–12. 10.1016/j.neuron.2008.11.023 19186163

[87] Husserl E The Basic Problems of Phenomenology: From the Lectures, Winter Semester, 1910–1911. Dordrecht: Springer (2006).

[88] Zahavi D Empathy, embodiment and interpersonal understanding: from Lipps to Schutz. Inquiry. (2010) 53:285–306. 10.1080/00201741003784663

[89] Gallese V Mirror neurons, embodied simulation, and the neural basis of social identification. Psychoanal Dialog. (2009) 19/5:519–36. 10.1080/10481880903231910

[90] Chelstrom E Gurwitsch the role of emotion in collective intentionality. In: Szanto T Moran D, editors. The Phenomenology of Sociality Discovering the 'We'. London: Routledge (2015). p. 248–62.

[91] Szanto T Moran D Edith Stein. In: Zalta EN, editor. Stanford Encyclopedia of Philosophy. (2020).

[92] Brosch T Scherer KR Grandjean D Sander D. The impact of emotion on perception, attention, memory, and decision-making. Swiss Med Wkly. (2013) 143:w13786. 10.4414/smw.2013.13786 23740562

[93] Flavell JH Perspectives on perspective taking. In: Beilin H Pufall PB, editors. The Jean Piaget Symposium Series: Vol. 14 Piaget's Theory: Prospects and Possibilities. Hillsdale, NJ: Erlbaum (1992). p. 107–39.

[94] Fuchs T Empathy, group identity, and the mechanisms of exclusion: an investigation into the limits of empathy. Topoi. (2019) 38:239–50. 10.1007/s11245-017-9499-z

[95] Varela FJ Present-time consciousness. J Conscious Stud. (1999) 6:111–40.

[96] Han S. Neurocognitive basis of racial ingroup bias in empathy. Trends Cogn Sci. (2018) 22:400–21. 10.1016/j.tics.2018.02.013 29563059

[97] Decety J Yang CY Cheng Y. Physicians down-regulate their pain empathy response: an event-related brain potential study. Neuroimage. (2010) 50:1676–82. 10.1016/j.neuroimage.2010.01.025 20080194

[98] Li W Han S. Perspective taking modulates event-related potentials to perceived pain. Neurosci Lett. (2010) 469:328–32. 10.1016/j.neulet.2009.12.021 20026179

[99] Ibáñez A Hurtado E Lobos A Escobar J Trujillo N Baez S. Subliminal presentation of other faces. (but not own face) primes behavioral and evoked cortical processing of empathy for pain. Brain Res. (2011) 1398:72–85. 10.1016/j.brainres.2011.05.014 21624566

[100] Sheng F Han S. Manipulations of cognitive strategies and intergroup relationships reduce the racial bias in empathic neural responses. Neuroimage. (2012) 61:786–97. 10.1016/j.neuroimage.2012.04.028 22542636

[101] Vistoli D Brunet-Gouet E Baup-Bobin E Hardy-Bayle MC Passerieux C. Anatomical and temporal architecture of theory of mind: a MEG insight into the early stages. Neuroimage. (2011) 54:1406–14. 10.1016/j.neuroimage.2010.09.015 20850554

[102] Bögels S Barr DJ Garrod S Kessler K. Conversational interaction in the scanner: mentalizing during language processing as revealed by MEG. Cereb Cortex. (2015) 25:3219–34. 10.1093/cercor/bhu116 24904076 PMC4537451

[103] Ferguson HJ Cane JE Douchkov M Wright D. Empathy predicts false belief reasoning ability: evidence from the N400. Soc Cogn Affect Neurosci. (2015) 10:848–55. 10.1093/scan/nsu131 25326041 PMC4448031

[104] Cikara M Van Bavel JJ. The neuroscience of intergroup relations: an integrative review. Perspect Psychol Sci. (2014) 9:245–74. 10.1177/1745691614527464 26173262

[105] De Dreu CKW Kret ME. Oxytocin conditions intergroup relations through upregulated in-group empathy, cooperation, conformity, and defense. Biol Psychiatry. (2016) 79:165–73. 10.1016/j.biopsych.2015.03.020 25908497

[106] Tajfel H Turner J An integrative theory of intergroup conflict. In: Austin WG Worchel S, editors. The Social Psycholgy of Intergroup Relations. Monterey, CA: Brooks & Cole (1979). p. 33–47.

[107] Hein G Silani G Preuschoff K Batson CD Singer T. Neural responses to ingroup and outgroup members' suffering predict individual differences in costly helping. Neuron. (2010) 68:149–60. 10.1016/j.neuron.2010.09.003 20920798

[108] Levy J Goldstein A Feldman R. Perception of social synchrony induces mother-child gamma coupling in the social brain. Soc Cogn Affect Neurosci. (2017) 12:1036–46. 10.1093/scan/nsx032 28402479 PMC5490671

[109] Pino MC Mazza M. The use of “Literary Fiction” to promote mentalizing ability. PLoS ONE. (2016) 11:e0160254. 10.1371/journal.pone.0160254 27490164 PMC4973931

[110] Hasson Y Shcori-Eyal N Daniel L Hasler BS Levy J Friedman D. The enemy's gaze: immersive virtual environments enhance peace promoting attitudes and emotions in violent intergroup conflicts. PLoS ONE. (2019) 14:e0222342. 10.1371/journal.pone.0222342 31509584 PMC6738917

[111] Influs M Pratt M Masalha S Zagoory-Sharon O Feldman R. A social neuroscience approach to conflict resolution: dialogue intervention to Israeli and Palestinian youth impacts oxytocin and empathy. Soc Neurosci. (2018) 14:378–89. 10.1080/17470919.2018.1479983 29799332

[112] Influs M Masalha S Zagoory-Shaon O Feldman R. Dialogue intervention to youth amidst intractable conflict attenuates stress response to outgroup. Horm Behav. (2019) 110:68–76. 10.1016/j.yhbeh.2019.02.013 30807738

[113] Amodio MD Cikara M The social neuroscience of prejudice. Ann Rev Psychol. (2021) 72 10.1146/annurev-psych-010419-050928 32946320

